# Barriers and facilitators to the dissemination of DECISION+, a continuing medical education program for optimizing decisions about antibiotics for acute respiratory infections in primary care: A study protocol

**DOI:** 10.1186/1748-5908-6-3

**Published:** 2011-01-07

**Authors:** Anne-Sophie Allaire, Michel Labrecque, Anik Giguère, Marie-Pierre Gagnon, Jeremy Grimshaw, France Légaré

**Affiliations:** 1 Research Center of Centre Hospitalier Universitaire de Québec, Hospital St-François D'Assise, Knowledge Transfer an Health Technology Assessment Research Group, 10 rue de l'Espinay, Québec, QC, G1L 3L5, Canada; 2 Department of Family Medicine and Emergency Medicine, Université Laval, Pavillon Ferdinand-Vandry, 1050 avenue de la Médecine, Québec, QC, G1V 0A6, Canada; 3 Faculty of Nursing, Université Laval, Pavillon Ferdinand-Vandry, 1050 avenue de la Médecine, Québec, QC, G1V 0A6, Canada; 4 Clinical Epidemiology Program, Ottawa Health Research Institute and Department of Medicine, University of Ottawa, 1053 Carling Avenue, Administration Building, Room 2-017, Ottawa ON K1Y 4E9, Canada

## Abstract

**Background:**

In North America, acute respiratory infections are the main reason for doctors' visits in primary care. Family physicians and their patients overuse antibiotics for treating acute respiratory infections. In a pilot clustered randomized trial, we showed that DECISION+, a continuing medical education program in shared decision making, has the potential to reduce the overuse of antibiotics for treating acute respiratory infections. DECISION+ learning activities consisted of three interactive sessions of three hours each, reminders at the point of care, and feedback to doctors on their agreement with patients about comfort with the decision whether to use antibiotics. The objective of this study is to identify the barriers and facilitators to physicians' participation in DECISION+ with the goal of disseminating DECISION+ on a larger scale.

**Methods/design:**

This descriptive study will use mixed methods and retrospective and prospective components. All analyses will be based on an adapted version of the Ottawa Model of Research Use. First, we will use qualitative methods to analyze the following retrospective data from the pilot study: the logbooks of eight research assistants, the transcriptions of 15 training sessions, and 27 participant evaluations of the DECISION+ training sessions. Second, we will collect prospective data in semi-structured focus groups composed of family physicians to identify barriers and facilitators to the dissemination of a future training program similar to DECISION+. All 39 family physicians exposed to DECISION+ during the pilot project will be eligible to participate. We will use a self-administered questionnaire based on Azjen's Theory of Planned Behaviour to assess participants' intention to take part in future training programs similar to DECISION+.

**Discussion:**

Barriers and facilitators identified in this project will guide modifications to DECISION+, a continuing medical education program in shared decision making regarding the use of antibiotics in acute respiratory infections, to facilitate its dissemination in primary care on a large scale. Our results should help continuing medical educators develop a continuing medical education program in shared decision making for other clinically relevant topics. This will help optimize clinical decisions in primary care.

## Background

In 2003, the province of Québec, Canada inaugurated a provincial public health policy to strengthen residents' capacity to make decisions regarding their own health [1]. Grounded in patient-centered care and scheduled to run from 2003 to 2012, this health policy aims to encourage patients to become more proactive regarding their own health and to exert full autonomy when making health-related decisions.

Shared decision making (SDM) could be a way to operationalize the strategy [2]. SDM can be defined as decisions that are shared by doctors and patients and that are informed not only by the best evidence on risks and benefits, but also by patients' personal characteristics and values. A systematic review of patient decision aids (also known as SDM programs) concludes that the aids help improve patients' awareness of their options and understanding of the benefits and disadvantages of each option [3]. This makes it easier for patients to make a decision and consequently increases patients' participation in the decision-making process.

In spite of the growing interest in SDM, few health professionals practice it, and only a few studies on implementing SDM in clinical settings exist [4]. A way to increase the practice of SDM might be to train professionals to identify decision points, convey the benefits and the risks of the options to their patients, and help the patients clarify their values and preferences [5-8].

Continuing medical education (CME) can be considered as an important knowledge translation intervention because of its potential to promote clinicians' adoption of best practices, including the practices needed for SDM to occur in primary care [9]. The DECISION+ training program is a CME program designed to improve family physicians' knowledge and skills in SDM, in order to optimize the prescription of antibiotics for acute respiratory infections (ARIs) [10]. ARIs are the principal reason for medical consultations in primary care in North America [11]. Although the effectiveness of antibiotics in treating most ARIs is limited, physicians nonetheless prescribed antibiotics for 56% of adult ARIs and 86% of child ARIs in the United States from 1995 to 2002 [12,13]. The probabilistic outcomes of treatments for ARIs and the trade-offs between the benefits and the harms of antibiotics make it difficult to choose the best course of action. Because of this uncertainty, patients should be invited to take part in deciding whether to use antibiotics for their ARI, and should go through a process of SDM with their physician if they desire. DECISION+ trains family physicians in SDM and teaches them ways to share with their patients the decision whether to prescribe antibiotics for the patient's ARI.

A pilot study conducted in 2007 evaluated the feasibility and acceptability of DECISION+ in family medicine clinics [14]. Following the intervention, the physicians who undertook DECISION+ training program prescribed fewer antibiotics. The reduction was not statistically significant, probably because the pilot study was underpowered [10]. However, only five of 24 eligible medical clinics agreed to participate in the pilot study and 25% of the 39 family physicians enrolled in the study did not complete the DECISION+ program [15]. Therefore, before disseminating DECISION+, it is essential not only to perform a larger trial but also to explore the barriers and facilitators to physicians' participation.

Identifying barriers and facilitators is a key step to implementing CME programs successfully in clinical practice [16]. A systematic review of studies published in 2004 and 2005 on barriers and facilitators to the implementation of SDM in clinical practice identified physicians' lack of time as the main obstacle [17]. However, of the 38 articles covered by the review, none studied the barriers and facilitators to the implementation or dissemination of CME programs in SDM. In a recent Cochrane systematic review of interventions to improve healthcare professionals' practice of SDM [4], the only study that showed a favourable impact of a coaching program with nurses, also assessed barriers and facilitators to healthcare professionals' practice of SDM but not to its SDM training program [8].

Consequently, the objective of this study is to identify barriers and facilitators to physicians' participation in the DECISION+ training program with the goal of disseminating DECISION+ on a larger scale.

## Conceptual framework

**Figure 1:**
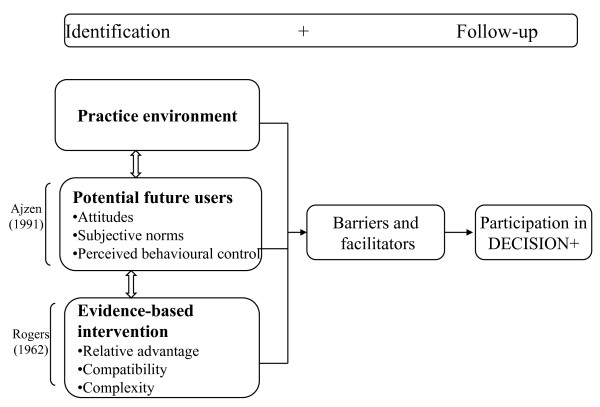
**Conceptual framework for the study of barriers and facilitators to the dissemination of DECISION+. **Adapted from the Ottawa Model of Research Use (Logan and Graham, 1998)

The project proposed by this protocol is based on the Ottawa Model of Research Use's theory of the mechanisms of planned change [18-20] (Figure 1). The Ottawa Model identifies three principal steps to evaluate how practitioners' use of research changes their behaviour: assessing barriers and facilitators related to practitioners' use of research; developing and monitoring interventions tailored to those barriers and facilitators; and evaluating outcomes. Assessing barriers and facilitators to practitioners' use of research requires investigating the relationship between the nature of practitioners' working environment, the characteristics of the intervention's potential future users, and the intervention in question (in this case, DECISION+). Data collection and analysis for this project will be guided by these three central elements of knowledge transfer and evidence-based innovations.

First, to assess barriers and facilitators related to the practice environment, we will gather descriptive data on potential external influences (*e.g.*, the infrastructure that houses the family medicine groups (FMGs) whose personnel participated in our pilot study, the number of doctors and nurses who work there) and the socioeconomic characteristics of the FMG's clientele and surrounding area.

Second, we will use Ajzen's Theory of Planned Behaviour to investigate barriers and facilitators related to potential future users [21]. Ajzen's theory has been shown to be useful for studying changes in the behaviour of health professionals [22]. According to the Theory of Planned Behaviour, intention is the immediate determinant of behaviour. It is driven by the will of the individual and is modulated through three direct variables: attitude, subjective norms, and perceived behavioural control. Attitude is the subject's favourable disposition to adopt a given behaviour. It derives from the consequences of the behaviour and from positive and negative judgments about these consequences. Subjective norms are the individual's belief that another person or a group thinks that he or she should or should not perform the behaviour [21]. Perceived behavioural control is the belief that a given factor will increase or reduce the difficulty of performing the behaviour. This last variable has been shown to be the main obstacle to adopting a behaviour [21].

Third, our study of the barriers and facilitators of the intervention will be inspired by Rogers's Diffusion of Innovation Theory [23]. According to Rogers, five elements determine the diffusion of an intervention: relative advantage, compatibility, complexity, testability, and observability. Only the first three of these elements have been shown to influence the adoption of an intervention [24] and consequently, only those three will be retained here. Relative advantage refers to the perception that an intervention is better than current practice. Compatibility measures the perception that an intervention is consistent with existing values, past experiments, social practices, and user standards. Finally, complexity refers to the perception that an intervention is difficult to include, understand and use.

## Methods/design

### Study design

This descriptive study will use mixed methods and include two components. First, retrospective data from the pilot DECISION+ trial (logbooks, written evaluations, and transcripts of audio recordings) will be qualitatively analyzed to identify barriers and facilitators to the dissemination of DECISION+.

Second, 18 months after the completion of the pilot project, we will collect prospective data from family physicians from the FMGs who participated in the pilot project. We will conduct semi-structured focus groups to gather the physicians' comments on the program. We will also distribute a self-administered questionnaire based on Azjen's Theory of Planned Behaviour to evaluate physicians' intention to take part in training programs similar to DECISION+ in future.

### Participants and recruitment strategy

The study population will consist of the 39 family physicians from the five FMGs who took part in our DECISION+ pilot study in Québec, Canada, in 2007 and 2008 [10]. We will begin by contacting the person in charge of the CME activities in each FMG (the contact person) to plan a meeting during which the researchers can share the results of pilot study with the physicians who participated in the pilot. During this presentation, the researchers will collect physicians' comments on their participation in the pilot. The contact person will be in charge of advertising the meeting and inviting physicians to attend. Before the meeting, the focus group methodology will be pretested and modified as necessary.

### Data collection

The retrospective data consists of recordings of the 15 training workshops conducted in the five FMGs, written evaluations of the workshops by 27 of the 39 original participants, and the eight logbooks kept by the research assistants.

For prospective data, we will organize five focus groups with the family physicians who participated in our pilot study. By operating with focus groups, we hope to maximize physicians' limited availability [25]. Because the lack of time for research is a major barrier to studying clinicians' behaviour, each focus group will be limited to a one-hour session. This may not allow sufficient time to collect all data. We are conscious of this limitation and will structure and standardize our data collection process to accommodate the time allotted. All sessions will be conducted within the same interview framework (Additional file 1).

One of the principal researchers of the pilot project (ML) will moderate the focus groups. One observer (ASA) will take notes on the process and the discussion. This will facilitate the recognition of participants during the transcription of the discussions.

The focus groups will assess participants' perceived barriers and facilitators to take part in a training program similar to DECISION+ within the following year. This program will address clinical topics relevant to participants' practice, other than the use of antibiotics to treat ARIs. At the end of the focus group, the participants will be invited to complete a short quantitative questionnaire that includes four questions on their sociodemographic characteristics and 11 questions based on the Theory of Planned Behaviour that assess their intention to participate in a future CME program similar to DECISION+.

Information on the number of doctors and nurses working in each FMG will be collected from the medical secretary of each organization. To help make the study findings transferable and reliable, a research team member (ASA) will complete a logbook in which she will note any environmental observations (*e.g.*, the type and location of the FMG), data about the participants (their number, their reactions), and information on any events that occur during the course of the interview. She will also note all modifications to the project and markers of its evolution.

### Data analysis

For the retrospective component of the project, a research team member will perform a qualitative analysis of all the material produced during the pilot study. S/he will use QSR NVivo 8 (QRS International Pty Ltd., Australia) to extract and regroup words, expressions, or short sentences with a view to analyzing the barriers and facilitators to physicians' participating in DECISION+. The member will produce an analytical report of the material.

For the prospective component of the project, we will record and transcribe all focus group discussions and carry out a thematic analysis using QSR NVivo 8. After having read and re-read the transcripts, we will apply an initial open code using deductive analysis based on the conceptual framework (Figure 1). We will initially cluster the barriers and facilitators to the use of research within the three central elements of knowledge transfer and evidence-based innovation, namely, the practice environment, potential future users, and the intervention. Thereafter, we will conduct an inductive analysis to identify emergent topics. Data will be coded and analyzed by a single person. To ensure the consistency of the qualitative process, preliminary results and the coding scheme will be discussed with the two members of the research team who are practicing family physicians (FL, ML). This will allow us to corroborate the code while protecting the code from being changed as a result of being applied by different people.

We will compare the phenomena observed to emphasize a common tangent. We will create tree structures and matrices for the analysis.

We will use descriptive statistics to analyze the sociodemographic variables, physicians' participation in the focus groups and pilot project, observational data, and answers to our questions based on the Theory of Planned Behaviour. We will use Pearson's correlation coefficient to assess the relationship between the variables from the questionnaire.

Triangulating the data from the retrospective and the prospective components of the study will enable us to suggest modifications to DECISION+.

## Discussion

Our findings will allow us to adapt and modify the DECISION+ program in order to facilitate its dissemination in the practice of primary care. CME is an important knowledge translation intervention that has the potential to promote the most effective practices. By targeting the barriers and the facilitators identified by the participants in the original DECISION+ pilot study, our study will allow us to propose modifications to the program so that it is better disseminated on a larger scale.

It is possible that the SDM skills that physicians acquire during DECISION+ can be extended to other health problems encountered in primary care. This study should help CME producers develop CME programs in SDM for other clinically relevant topics. This would help optimize clinical decisions in primary care.

In short, this study will improve our understanding not only of how DECISION+ can be disseminated, but also how to disseminate other CME activities regarding the practice of SDM in primary care.

## Competing interests

The authors declare that they have no competing interests.

## Authors' contributions

ASA, FL and ML conceived the study and validated the methods. ASA wrote the first draft and revised the protocol. FL, ML, AG, JG each reviewed and modified various versions of the protocol. All authors have read and approved the final manuscript.

## Supplementary Material

Additional file 1 **Focus group interview framework. **Questions ask during the focus group. -Presentation of the results of the DECISION+ pilot project to participants and invitation to ask questions. -Physicians' perceptions of the innovations of DECISION+ compared to other CME programs. -Physicians' perceptions of factors that could encourage them to take part in a program similar to DECISION+. -Physicians' perceptions of factors that could discourage them from taking part in a program similar to DECISION+. -Any suggestions or comments to improve participation in a program similar to DECISION+ Click here for file
