# Supplementary material for: Barriers and facilitators to the dissemination of DECISION+, a continuing medical education program for optimizing decisions about antibiotics for acute respiratory infections in primary care: A study protocol
Source: Implement Sci. 2011 Jan 7;6:3. doi: 10.1186/1748-5908-6-3 (PMC3023690; doi:10.1186/1748-5908-6-3)
Supplement: Additional file 1 — Focus group interview framework. Questions ask during the focus group. -Presentation of the results of the DECISION+ pilot project to participants and invitation to ask questions. -Physicians' perceptions of the innovations of DECISION+ compared to other CME programs. -Physicians' perceptions of factors that could encourage them to take part in a program similar to DECISION+. -Physicians' perceptions of factors that could discourage them from taking part in a program similar to DECISION+. -Any suggestions or comments to improve participation in a program similar to DECISION+ [file 1748-5908-6-3-S1.DOC]

Additional files

Additional file 1
Title: Focus group interview framework
Description: Questions ask during the focus group.

-Presentation of the results of the DECISION+ pilot project to participants and invitation to ask questions

-Physicians’ perceptions of the innovations of DECISION+ compared to other CME programs

-Physicians’ perceptions of factors that could encourage them to take part in a program similar to DECISION+

-Physicians’ perceptions of factors that could discourage them from taking part in a program similar to DECISION+

-Any suggestions or comments to improve participation in a program similar to DECISION+
